# (7a*S*)-(–)-Dimeth­yl(1-oxido-3-oxo-5,6,7,7a-tetra­hydro-3*H*-pyrrolizin-2-yl)sulfonium

**DOI:** 10.1107/S1600536812003601

**Published:** 2012-02-17

**Authors:** Leonardo Gutiérrez-Lazcano, Joel L. Terán, Jorge R. Juárez, Marcos Flores-Alamo, Angel Mendoza

**Affiliations:** a Centro de Química, Instituto de Ciencias, Benemérita Universidad Autónoma de Puebla, Puebla, Pue., Mexico; b Facultad de Química, Universidad Nacional Autónoma de México, 04510 México, DF, Mexico

## Abstract

In the zwitterionic title compound, C_9_H_13_NO_2_S, the pyrrolidine heterocycle adopts an envelope conformation (with the C atom in the 7-position as the flap). The negative charge is delocalized over the two carbonyl groups and the C atom connecting them. The positive charge is located on the S atom. Two inter­molecular C—H⋯O inter­actions are observed. The molecular geometry at the S atom is trigonal pyramidal.

## Related literature

For background to the synthesis of chiral non-racemic zwitterionic compounds, see: Zang *et al.* (2008 ▶); Kappe *et al.* (1983 ▶); Palillero *et al.* (2009 ▶). For the biological activity of related structures, see: Basco *et al.* (1994 ▶); Koruznjak *et al.* (2003 ▶). For puckering parameters, see: Cremer & Pople (1975 ▶).
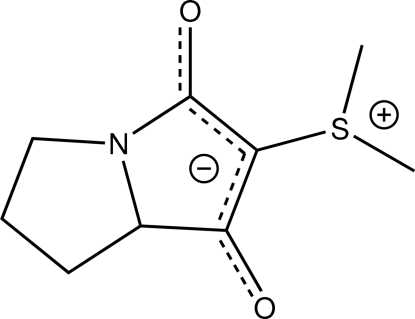


## Experimental

### 

#### Crystal data

C_9_H_13_NO_2_S
*M* *_r_* = 199.26
Orthorhombic, 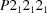

*a* = 5.8761 (3) Å
*b* = 9.0858 (5) Å
*c* = 17.7107 (9) Å
*V* = 945.56 (9) Å^3^
*Z* = 4
Mo *K*α radiation
μ = 0.31 mm^−1^
*T* = 130 K
0.46 × 0.33 × 0.07 mm

#### Data collection

Oxford Xcalibur Atlas Gemini diffractometer
Absorption correction: analytical (*CrysAlis PRO*; Oxford Diffraction, 2009 ▶) *T*_min_ = 0.895, *T*_max_ = 0.976
6356 measured reflections
1873 independent reflections
1736 reflections with *I* > 2σ(*I*)
*R*_int_ = 0.037

#### Refinement

*R*[*F* ^2^ > 2σ(*F* ^2^)] = 0.027
*wR*(*F* ^2^) = 0.065
*S* = 1.04
1873 reflections
120 parameters
H-atom parameters constrained
Δρ_max_ = 0.20 e Å^−3^
Δρ_min_ = −0.26 e Å^−3^
Absolute structure: Flack (1983 ▶), with 758 Friedel pairs
Flack parameter: −0.07 (7)

### 

Data collection: *CrysAlis PRO* (Oxford Diffraction, 2009 ▶); cell refinement: *CrysAlis PRO*; data reduction: *CrysAlis RED* (Oxford Diffraction, 2002 ▶); program(s) used to solve structure: *SHELXS97* (Sheldrick, 2008 ▶); program(s) used to refine structure: *SHELXL97* (Sheldrick, 2008 ▶); molecular graphics: *ORTEP-3 for Windows* (Farrugia, 1997 ▶); software used to prepare material for publication: *WinGX* (Farrugia, 1999 ▶).

## Supplementary Material

Crystal structure: contains datablock(s) global, I. DOI: 10.1107/S1600536812003601/bt5793sup1.cif

Structure factors: contains datablock(s) I. DOI: 10.1107/S1600536812003601/bt5793Isup2.hkl

Supplementary material file. DOI: 10.1107/S1600536812003601/bt5793Isup3.cml

Additional supplementary materials:  crystallographic information; 3D view; checkCIF report

## Figures and Tables

**Table 1:** Hydrogen-bond geometry (Å, °)

*D*—H⋯*A*	*D*—H	H⋯*A*	*D*⋯*A*	*D*—H⋯*A*
C4—H4⋯O2^i^	1.00	2.55	3.4145 (19)	145
C7—H7*B*⋯O1^ii^	0.99	2.59	3.570 (2)	173

Symmetry codes: (i) 
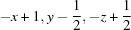
; (ii) 
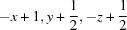
.

**Fig. 1.**
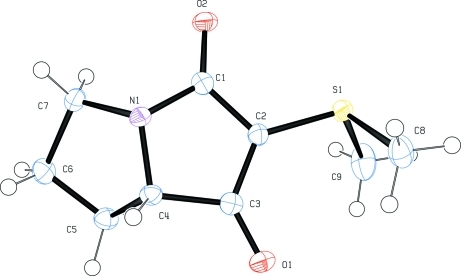
The molecular structure of title compound, with atom labels and 50% probability displacement ellipsoids for non-H atoms.

## supplementary crystallographic information

### Comment

The synthesis of chiral non racemic zwitterionic compounds is an original area of interest in organic chemistry (Zang *et al.*, 2008; Kappe *et al.*, 1983) because they are useful intermediates for the synthesis of diverse interesting nitrogen heterocyclic compounds (Palillero *et al.*, 2009) with interesting biological properties (Basco *et al.*, 1994; Koruznjak *et al.*, 2003).

In the title zwitterionic compound, C_19_H_13_NO_2_S, the chiral centre shows an *S* configuration, and the five membered pyrrolidine heterocycle shows an envelope conformations on C5 with puckering parameters (Cremer & Pople, 1975) φ_2_ = 258.4 (3)° and q_2_ = 0.4038 (19) Å. The five membered ring N1/C1/C2/C3/C4 shows a twist conformation on N1—C1 with puckering parameters φ_2_ = 0.0903 (18)° and q_2_ = 22.4 (12) Å. The bond distances of C1—O2 [1.235 (2) Å] and C3—O1 [1.238 (2) Å] are similar as in related systems which were previously reported. The C2—C3 bond distance [1.406 (2) Å] has the same length as an aromatic bond and C2—C1 [1.435 (2) Å] is shorter than a typical *sp*^3^—*sp*^3^ bond distance. This suggests, that the negative charge is delocalized on the O1/C3/C2/C2/O2 system. Two intermolecular weak interactions C4—H4···O2 (3.412 (2) Å) and C7—H7B···O1 (3.570 (2) Å) are observed.

### Experimental

The title compound, was obtained by an intramolecular cyclization reaction of (*S*)-(-)-[2-(2-Methoxycarbonyl-pyrrolidin-1-yl)-2-oxo-ethyl]-dimethyl-sulfonium; bromide (1 mmol), which was dissolved in CH_3_CN (10 ml), treated with KOH (1.2 mmol) and stirred for 2 h at room temperature. The resulting mixture was concentrated in vacuum and dissolved in ethyl acetate, filtered and concentrated giving the desired compound in 98%. Crystals were obtained from an ethyl acetate/diethyl ether solution; m.p. 110–112 °C, [α]_D_= -13.4 (*c* 1.0, CH_2_Cl_2_). IR (KBr) 3447, 1655, 1591, 1372 cm^-1. 1^H NMR (400 MHz, CDCl_3_) d(p.p.m., *J*Hz): 1.51 (m, 1H), 2.05 (m, 3H), 2.99 (s, 3H), 3.01 (s, 3H), 3.12 (m, 1H), 3.57 (td, *J* = 7.8, 11.0 Hz, 1H), 3.85 (dd, *J* = 7.24, 9.28 Hz, 1H). HRMS (FAB+): Calcd for C_9_H_13_NO_2_S: 199.0667. Found: 199.0665.

### Refinement

H atoms were placed in geometrically idealized positions and refined as riding on their parent atoms, with C—H distances fixed to 0.960 (methyl CH_3_) and 0.980 Å (methine CH) and with *U*_iso_(H) = 1.5U_eq_(methyl C) or 1.2U_eq_(C).

### Crystal data

**Table:**

C_9_H_13_NO_2_S	*D*_x_ = 1.406 Mg m^−^^3^
*M**_r_* = 199.26	Mo *K*α radiation, λ = 0.71073 Å
Orthorhombic, *P*2_1_2_1_2_1_	Cell parameters from 4129 reflections
*a* = 5.8761 (3) Å	θ = 3.5–26.0°
*b* = 9.0858 (5) Å	µ = 0.31 mm^−^^1^
*c* = 17.7107 (9) Å	*T* = 130 K
*V* = 945.56 (9) Å^3^	Plate, colourless
*Z* = 4	0.46 × 0.33 × 0.07 mm
*F*(000) = 424	

### Data collection

**Table:**

Oxford Xcalibur Atlas Gemini diffractometer	1873 independent reflections
Graphite monochromator	1736 reflections with *I* > 2σ(*I*)
Detector resolution: 10.4685 pixels mm^-1^	*R*_int_ = 0.037
ω scans	θ_max_ = 26.1°, θ_min_ = 3.7°
Absorption correction: analytical (*CrysAlis PRO*; Oxford Diffraction, 2002)	*h* = −7→7
*T*_min_ = 0.895, *T*_max_ = 0.976	*k* = −10→11
6356 measured reflections	*l* = −18→21

### Refinement

**Table:**

Refinement on *F*^2^	Secondary atom site location: difference Fourier map
Least-squares matrix: full	Hydrogen site location: inferred from neighbouring sites
*R*[*F*^2^ > 2σ(*F*^2^)] = 0.027	H-atom parameters constrained
*wR*(*F*^2^) = 0.065	*w* = 1/[σ^2^(*F*_o_^2^) + (0.0385*P*)^2^] where *P* = (*F*_o_^2^ + 2*F*_c_^2^)/3
*S* = 1.04	(Δ/σ)_max_ < 0.001
1873 reflections	Δρ_max_ = 0.20 e Å^−^^3^
120 parameters	Δρ_min_ = −0.26 e Å^−^^3^
0 restraints	Absolute structure: Flack (1983), with 758 Friedel pairs
Primary atom site location: structure-invariant direct methods	Flack parameter: −0.07 (7)

### Special details

**Table:**

Geometry. All s.u.'s (except the s.u. in the dihedral angle between two l.s. planes) are estimated using the full covariance matrix. The cell s.u.'s are taken into account individually in the estimation of s.u.'s in distances, angles and torsion angles; correlations between s.u.'s in cell parameters are only used when they are defined by crystal symmetry. An approximate (isotropic) treatment of cell s.u.'s is used for estimating s.u.'s involving l.s. planes.
Refinement. Refinement of *F*^2^ against ALL reflections. The weighted *R*-factor *wR* and goodness of fit *S* are based on *F*^2^, conventional *R*-factors *R* are based on *F*, with *F* set to zero for negative *F*^2^. The threshold expression of *F*^2^ > 2σ(*F*^2^) is used only for calculating *R*-factors(gt) *etc*. and is not relevant to the choice of reflections for refinement. *R*-factors based on *F*^2^ are statistically about twice as large as those based on *F*, and *R*-factors based on ALL data will be even larger.

### Fractional atomic coordinates and isotropic or equivalent isotropic displacement parameters (Å^2^)

**Table:**

	*x*	*y*	*z*	*U*_iso_*/*U*_eq_	
S1	0.72287 (7)	0.59085 (4)	0.01093 (2)	0.01852 (12)	
O1	0.6977 (3)	0.26576 (13)	0.09183 (7)	0.0293 (3)	
O2	0.4048 (2)	0.74463 (12)	0.13031 (6)	0.0229 (3)	
N1	0.4198 (2)	0.53624 (15)	0.20467 (8)	0.0172 (3)	
C1	0.4721 (3)	0.61719 (18)	0.14100 (9)	0.0172 (4)	
C2	0.6056 (3)	0.52340 (19)	0.09255 (9)	0.0188 (4)	
C3	0.6092 (3)	0.37738 (18)	0.11921 (10)	0.0193 (4)	
C4	0.4806 (3)	0.38002 (17)	0.19391 (9)	0.0173 (4)	
H4	0.5823	0.3464	0.2358	0.021*	
C5	0.2519 (3)	0.30179 (17)	0.19885 (10)	0.0216 (4)	
H5A	0.2702	0.1975	0.2138	0.026*	
H5B	0.1682	0.3069	0.1504	0.026*	
C6	0.1329 (3)	0.3908 (2)	0.26032 (10)	0.0228 (4)	
H6A	0.1871	0.3614	0.3111	0.027*	
H6B	−0.0341	0.3772	0.2579	0.027*	
C7	0.1984 (3)	0.55113 (19)	0.24287 (10)	0.0222 (4)	
H7A	0.0847	0.5982	0.2094	0.027*	
H7B	0.2125	0.6096	0.2898	0.027*	
C8	1.0123 (3)	0.5311 (2)	0.01195 (12)	0.0296 (4)	
H8A	1.0832	0.5535	−0.0368	0.044*	
H8B	1.0179	0.4248	0.0209	0.044*	
H8C	1.0946	0.5823	0.0523	0.044*	
C9	0.6187 (4)	0.4744 (3)	−0.06291 (11)	0.0370 (5)	
H9A	0.6929	0.5007	−0.1106	0.056*	
H9B	0.4538	0.4873	−0.0679	0.056*	
H9C	0.6523	0.3715	−0.0507	0.056*	

### Atomic displacement parameters (Å^2^)

**Table:**

	*U*^11^	*U*^22^	*U*^33^	*U*^12^	*U*^13^	*U*^23^
S1	0.0209 (2)	0.0186 (2)	0.0160 (2)	0.00209 (17)	0.00126 (16)	0.00138 (16)
O1	0.0388 (8)	0.0202 (6)	0.0290 (7)	0.0101 (6)	0.0091 (6)	0.0018 (5)
O2	0.0311 (7)	0.0156 (6)	0.0220 (6)	0.0036 (5)	0.0008 (6)	−0.0003 (5)
N1	0.0200 (8)	0.0147 (7)	0.0170 (7)	−0.0002 (6)	0.0002 (6)	−0.0023 (6)
C1	0.0173 (9)	0.0194 (9)	0.0150 (8)	−0.0035 (7)	−0.0038 (7)	−0.0014 (7)
C2	0.0205 (9)	0.0190 (8)	0.0169 (9)	0.0022 (8)	0.0023 (8)	0.0016 (7)
C3	0.0178 (9)	0.0193 (9)	0.0209 (9)	−0.0004 (7)	−0.0012 (7)	0.0003 (7)
C4	0.0176 (8)	0.0165 (8)	0.0179 (9)	0.0031 (7)	−0.0021 (7)	0.0013 (7)
C5	0.0213 (10)	0.0186 (8)	0.0250 (9)	−0.0018 (8)	−0.0005 (8)	−0.0005 (7)
C6	0.0166 (9)	0.0247 (10)	0.0272 (9)	−0.0060 (8)	0.0030 (7)	−0.0001 (8)
C7	0.0236 (10)	0.0210 (8)	0.0221 (9)	−0.0006 (8)	0.0067 (8)	−0.0029 (7)
C8	0.0191 (9)	0.0384 (10)	0.0314 (10)	0.0013 (8)	0.0018 (8)	0.0086 (9)
C9	0.0366 (13)	0.0563 (13)	0.0181 (10)	−0.0149 (11)	−0.0006 (9)	−0.0074 (10)

### Geometric parameters (Å, º)

**Table:**

S1—C2	1.7146 (17)	C5—H5A	0.99
S1—C8	1.7851 (18)	C5—H5B	0.99
S1—C9	1.7900 (19)	C6—C7	1.538 (3)
O1—C3	1.238 (2)	C6—H6A	0.99
O2—C1	1.238 (2)	C6—H6B	0.99
N1—C1	1.381 (2)	C7—H7A	0.99
N1—C7	1.473 (2)	C7—H7B	0.99
N1—C4	1.476 (2)	C8—H8A	0.98
C1—C2	1.442 (2)	C8—H8B	0.98
C2—C3	1.408 (2)	C8—H8C	0.98
C3—C4	1.524 (2)	C9—H9A	0.98
C4—C5	1.523 (2)	C9—H9B	0.98
C4—H4	1	C9—H9C	0.98
C5—C6	1.526 (2)		
			
C2—S1—C8	105.40 (9)	H5A—C5—H5B	109.3
C2—S1—C9	105.50 (9)	C5—C6—C7	104.11 (14)
C8—S1—C9	98.84 (11)	C5—C6—H6A	110.9
C1—N1—C7	121.50 (14)	C7—C6—H6A	110.9
C1—N1—C4	110.68 (13)	C5—C6—H6B	110.9
C7—N1—C4	111.17 (13)	C7—C6—H6B	110.9
O2—C1—N1	123.51 (15)	H6A—C6—H6B	109
O2—C1—C2	129.47 (15)	N1—C7—C6	103.08 (13)
N1—C1—C2	106.99 (14)	N1—C7—H7A	111.1
C3—C2—C1	111.43 (15)	C6—C7—H7A	111.1
C3—C2—S1	127.84 (13)	N1—C7—H7B	111.1
C1—C2—S1	120.61 (13)	C6—C7—H7B	111.1
O1—C3—C2	130.27 (16)	H7A—C7—H7B	109.1
O1—C3—C4	124.12 (15)	S1—C8—H8A	109.5
C2—C3—C4	105.60 (14)	S1—C8—H8B	109.5
N1—C4—C5	103.19 (13)	H8A—C8—H8B	109.5
N1—C4—C3	104.31 (13)	S1—C8—H8C	109.5
C5—C4—C3	118.71 (14)	H8A—C8—H8C	109.5
N1—C4—H4	110	H8B—C8—H8C	109.5
C5—C4—H4	110	S1—C9—H9A	109.5
C3—C4—H4	110	S1—C9—H9B	109.5
C4—C5—C6	101.43 (13)	H9A—C9—H9B	109.5
C4—C5—H5A	111.5	S1—C9—H9C	109.5
C6—C5—H5A	111.5	H9A—C9—H9C	109.5
C4—C5—H5B	111.5	H9B—C9—H9C	109.5
C6—C5—H5B	111.5		
			
C7—N1—C1—O2	34.5 (2)	S1—C2—C3—C4	179.92 (13)
C4—N1—C1—O2	167.62 (15)	C1—N1—C4—C5	−116.83 (15)
C7—N1—C1—C2	−143.55 (15)	C7—N1—C4—C5	21.34 (17)
C4—N1—C1—C2	−10.39 (18)	C1—N1—C4—C3	7.84 (18)
O2—C1—C2—C3	−168.74 (17)	C7—N1—C4—C3	146.01 (13)
N1—C1—C2—C3	9.1 (2)	O1—C3—C4—N1	176.79 (16)
O2—C1—C2—S1	7.6 (3)	C2—C3—C4—N1	−2.07 (18)
N1—C1—C2—S1	−174.58 (12)	O1—C3—C4—C5	−69.1 (2)
C8—S1—C2—C3	−52.18 (19)	C2—C3—C4—C5	112.01 (16)
C9—S1—C2—C3	51.81 (19)	N1—C4—C5—C6	−37.44 (16)
C8—S1—C2—C1	132.16 (15)	C3—C4—C5—C6	−152.13 (15)
C9—S1—C2—C1	−123.85 (16)	C4—C5—C6—C7	40.51 (17)
C1—C2—C3—O1	177.13 (18)	C1—N1—C7—C6	136.85 (15)
S1—C2—C3—O1	1.1 (3)	C4—N1—C7—C6	3.89 (18)
C1—C2—C3—C4	−4.1 (2)	C5—C6—C7—N1	−27.69 (17)

### Hydrogen-bond geometry (Å, º)

**Table:**

*D*—H···*A*	*D*—H	H···*A*	*D*···*A*	*D*—H···*A*
C4—H4···O2^i^	1.00	2.55	3.4145 (19)	145
C7—H7*B*···O1^ii^	0.99	2.59	3.570 (2)	173

Symmetry codes: (i) −*x*+1, *y*−1/2, −*z*+1/2; (ii) −*x*+1, *y*+1/2, −*z*+1/2.
